# Efficient generation of twin photons at telecom wavelengths with 2.5 GHz repetition-rate-tunable comb laser

**DOI:** 10.1038/srep07468

**Published:** 2014-12-19

**Authors:** Rui-Bo Jin, Ryosuke Shimizu, Isao Morohashi, Kentaro Wakui, Masahiro Takeoka, Shuro Izumi, Takahide Sakamoto, Mikio Fujiwara, Taro Yamashita, Shigehito Miki, Hirotaka Terai, Zhen Wang, Masahide Sasaki

**Affiliations:** 1 National Institute of Information and Communications Technology (NICT), 4-2-1 Nukui-Kitamachi, Koganei, Tokyo 184-8795, Japan; 2 University of Electro-Communications (UEC), 1-5-1 Chofugaoka, Chofu Tokyo 182-8585, Japan; 3 Sophia University, 7-1 Kioicho, Chiyoda-ku, Tokyo 102-8554, Japan; 4 National Institute of Information and Communications Technology (NICT), 588-2 Iwaoka, Kobe 651-2492, Japan; 5 Shanghai Institute of Microsystem and Information Technology, Chinese Academy of Sciences (CAS), 865 Changning Road, Shanghai 200050, China

## Abstract

Efficient generation and detection of indistinguishable twin photons are at the core of quantum information and communications technology (Q-ICT). These photons are conventionally generated by spontaneous parametric down conversion (SPDC), which is a probabilistic process, and hence occurs at a limited rate, which restricts wider applications of Q-ICT. To increase the rate, one had to excite SPDC by higher pump power, while it inevitably produced more unwanted multi-photon components, harmfully degrading quantum interference visibility. Here we solve this problem by using recently developed 10 GHz repetition-rate-tunable comb laser, combined with a group-velocity-matched nonlinear crystal, and superconducting nanowire single photon detectors. They operate at telecom wavelengths more efficiently with less noises than conventional schemes, those typically operate at visible and near infrared wavelengths generated by a 76 MHz Ti Sapphire laser and detected by Si detectors. We could show high interference visibilities, which are free from the pump-power induced degradation. Our laser, nonlinear crystal, and detectors constitute a powerful tool box, which will pave a way to implementing quantum photonics circuits with variety of good and low-cost telecom components, and will eventually realize scalable Q-ICT in optical infra-structures.

Since the first experimental realization of quantum teleportation^1^, many experiments with multiphoton entanglement have been demonstrated^2^,^3^, and currently expanded to eight photons, employing multiple SPDC crystals^4^,^5^. In order to increase the scale of entanglement further, the generation probability per SPDC crystal must be drastically improved without degrading the quantum indistinguishability of photons. Unfortunately, however, a dilemma always exists in SPDC: higher pump power is required for higher generation probability, while it degrades quantum interference visibility due to unwanted multi-pair emissions, leading to the increase of error rates in entanglement-based quantum key distribution (QKD)^6^ and photonic quantum information processing^7^.

When *n* SPDC sources are used, the 2*n*-fold coincidence counts (CC) can be estimated as


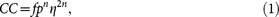


where *f* is the repetition rate of the pump laser, *p* is the generation probability of one photon pair per pulse (averaged photon pair number per pulse), *η* is the overall efficiency, which is the product of the collecting efficiency of the whole optical system and the detecting efficiency of the detectors. The *p* should be restricted to less than 0.1, so that the effect of unwanted multi-pair emissions can be negligible. So the pump power is tuned for *p* ≤ 0.1.

The value of *p* is not high in the conventional photon source. A standard technology is based on SPDC at visible and near infrared wavelengths using a BBO crystal pumped by the second harmonic of the femto-second laser pulses from a Ti Sapphire laser, whose repetition rate is 76 MHz^2^,^4^,^5^. In this case, the probability *p* had not been able to go beyond 0.06, because the second harmonic power was limited to 300–900 mW for a fundamental laser power of 1–3 W. Therefore, recent efforts have been focused on increasing the pump power^8^.

Recently the periodically poled KTP (PPKTP) crystals attract much attention because it can achieve *p* ~ 0.1 (0.6) at telecom wavelengths with a pump power of 80 (400) mW thanks to the quasi-phase matching (QPM) technique^9^. When waveguide structure is employed, *p* can be 10^3^ times higher than the bulk crystal^10^,^11^. Unfortunately, however, the constraint *p* ≤ 0.1 should be met in these cases too. The *η* is already maximized by careful alignment in laboratories, e.g., the typical *η* value is about 0.2–0.3^4^,^5^,^9^. Thus *p* and *η* have almost reach their maxima. The remaining effective way is to improve the repetition rate of the pump laser, *f*.

In this work, we demonstrate a novel photon source pumped by a recently developed repetition-rate-tunable comb laser in a range of 10–0.625 GHz^12^,^13^. This laser can operate in relatively low pulse energy, while keeping high average power, thanks to a high repetition rate. The low pulse energy would result in the reduction of the multiple-pair emission. At the same time a high counting rate would be expected owing to the high average power. The SPDC based on a group-velocity-matched PPKTP (GVM-PPKTP) crystal can achieve very high spectral purity of the constituent photons^14^,^15^. Furthermore, the photons are detected by the state-of-the-art superconducting nanowire single photon detectors (SNSPDs)^16^,^17^, which have a much higher efficiency than that of traditional InGaAs avalanche photodiode (APD).

## Experiment

**Figure 1:**
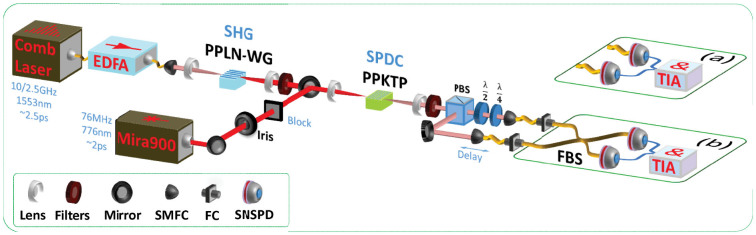
The Experimental setup. A comb laser with a 10–0.625 GHz tunable-repetition-rate at 1553 nm wavelength is amplified by a high-power erbium-doped fiber amplifier (EDFA), and frequency doubled by a 10-mm-long type-0 periodically poled lithium niobate wave guide (PPLN-WG, by NTT Electronics, polling period = 18.2 *μ*m). Then, after filtered by short-pass filters, the 776.5 nm laser light from second harmonic generation (SHG) pumps a 30-mm type-II periodically poled potassium titanyl phosphate crystal (PPKTP, by Raicol, poling period = 46.1 *μ*m, temperature = 47.8°C) for SPDC. The downconverted photons, the signal and idler, are filtered by long-pass filters, separated by a polarization beamsplitter (PBS), and coupled into single mode fibers (SMF) by couplers (SMFC). (a) The collected photons are directly detected by SNSPDs, which are connected to a time interval analyzer (TIA) for the measurement of Time of Arrival (ToA). (b) SMFs are connected to a fiber beamsplitter (FBS) by two fiber connectors (FC), for the test of Hong-Ou-Mandel dip. A half-wave plate and a quarter-wave plate are added to guarantee the signal and idler photons have the same polarization in FBS. For comparison, we also introduce a 76 MHz pump laser (Mira900, at 776.5 nm, around 2 ps). An iris is inserted for the 76 MHz laser to control the beam size, so as to make the coincidence counts comparable for both pump lasers.

**Figure 2:**
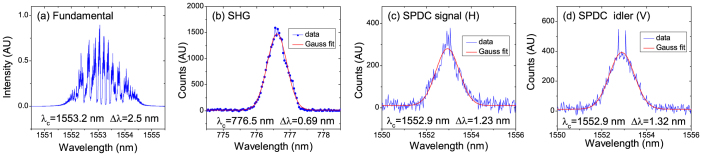
The spectra. (a) The spectra of the comb laser at 2.5 GHz repetition rates. (b) A typical spectrum of SHG, pumped by the fundamental laser. (c, d) Spectra of the signal (horizontal polarization) and idler (vertical polarization) photons generated in SPDC.

The experimental setup is shown in Fig. 1. The repetition rate of the comb laser used in this experiment has a long tunable range, and we mainly use the 2.5 GHz repetition rate, since the SHG laser power at 10 GHz repetition rate was not as stable as that at 2.5 GHz repetition rate. Fig. 2(a–d) show the spectra of the fundamental, the SHG and the SPDC photons. The performance with the 2.5 GHz source is evaluated in terms of a signal to noise ratio (SNR)^18^ and a Hong-Ou-Mandel (HOM) interference^19^, in comparison with the 76 MHz laser. The SNR test is carried out in the detecting configuration of Fig. 1(a), while the HOM test is performed in that of Fig. 1(b).

## Results

### 1: Signal to noise ratio test

**Figure 3:**
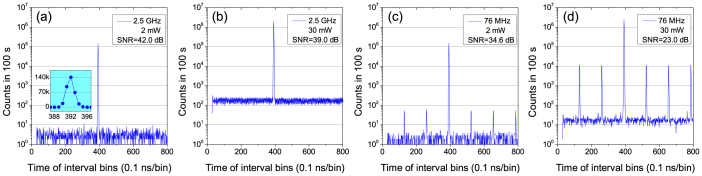
Time of Arrival (ToA) data. The data in each figure was accumulated in 100 seconds for 2.5 GHz and 76 MHz lasers at 2 mW and 30 mW. The peak-to-peak intervals are 0.4 ns in (a), (b) and 13.1 ns in (c), (d). The measured SNR values are 42.0 dB, 39.0 dB, 34.6 dB and 23.0 dB for (a)–(d), respectively. Inset in (a) is an enlarged view of the main peak in a linear scale.

The SNR can be approximated as the ratio of single pair emission rate over the double pair emission rate^18^. The SNR can be evaluated by Time of Arrival (ToA) data, which are shown in Fig. 3(a–d). Each data consists of the main peak and side peaks, and we define the peak counts as the value of each peak. The side peaks are not visible in Fig. 3(a, b) because the resolution of the detector system (~0.5 ns, as seen in the inset in (a)) is comparable to the pulse interval of 2.5 GHz laser (0.4 ns). So, we set the averaged maximal counts as the side peak values. The side peaks are recorded when a second SPDC occurs (photons detected in the stop channel) conditioned on a first SPDC occurs (photons detected in the start channel) at the main peaks. Therefore, the side peaks correspond to the rate of 2-pair components in SPDC, while the main peaks correspond to the rate of 1-pair plus 2-pair components in SPDC. So the SNR can be calculated in dB as
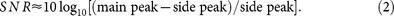


Note this definition is valid for lower pump power regime. See more discussions in the Supplementary Information. We did not include excess noise in the analysis, since the dark counts of the detectors were very low and other fluorescence was negligible in our experiment. The measured SNR are 42.0 dB, 39.0 dB, 34.6 dB, 23.0 dB for Fig. 3(a–d), respectively.

Theoretical calculation unveils that the SNR is proportional to the inverse of average photon numbers per pulse at a lower pump power^18^. This claim can be experimentally verified by comparing Fig. 3(c) and (d) with the 76 MHz laser. When the pump power increases from 2 mW to 30 mW, the SNR is decreased by 34.6–23.0 = 11.6 dB. It agrees well with the 30 mW/2 mW = 15 times (11.7 dB) increase of average photon number per pulse.

Next, we compare the result in Fig. 3(b) and (d), so as to confirm the validity of the definition for side peak values in Fig. 3(a) and (b). At 30 mW pump power, the coincidence counts are 48 kcps and 56 kcps for 2.5 GHz and 76 MHz laser, respectively, which can also be obtained by summing all the counts in a 2 ns coincidence window centered at the main peak in Fig. 3(b) and (d), and then dividing the 100 s accumulation time. Then the average photon numbers per pulse are estimated to be 0.00021 and 0.0079, correspondingly, using the method in ref 10, and considering the single counts of 150/165 kcps for 2.5 GHz laser and 178/189 kcps for 76 MHz laser. The average photon pair per pulse for the 2.5 GHz laser is 0.0079/0.00021 = 37.6 times (15.8 dB) lower than that of the 76 MHz laser. Recall the SNR difference between 2.5 GHz and 76 MHz of 39.0–23.0 = 16.0 dB. This consistency verifies the validity of the definition for side peak values in Fig. 3(a) and (b).

Finally, we estimate the SNR values for the case of the 2.5 GHz laser at 2 mW in Fig. 3(a). The SNR at 2 mW should ideally increase by 11.7 dB (15 times), from 39.0 dB (30 mW in Fig. 3(b)) to 50.7 dB. Actually, however, the measured SNR is only 42.0 dB. This discrepancy is mainly due to the dark counts by the detectors and the accidental counts by stray photons.

### 2: Hong-Ou-Mandel interference test

**Figure 4:**
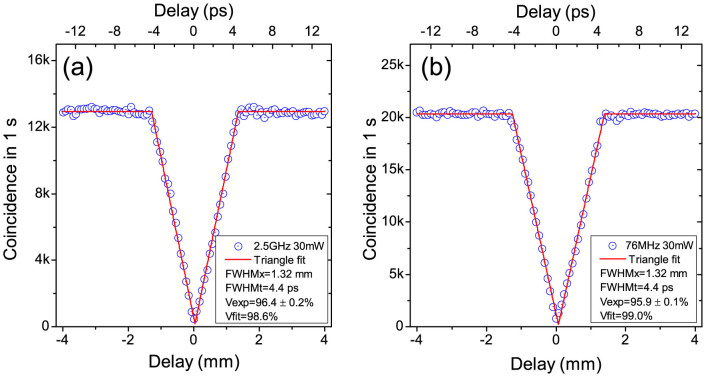
Hong-Ou-Mandel (HOM) dip. HOM dip for 2.5 GHz (a) and 76 MHz (b) repetition rate lasers with pump power of 30 mW, fitted with triangle shape function.

We then carried out the HOM interference test to evaluate the performance of a twin photon source. We firstly worked with 30 mW pump power for 2.5 GHz and 76 MHz repetition rate lasers, and achieved raw visibilities of 96.4 ± 0.2% and 95.9 ± 0.1%, respectively, as shown in Fig. 4(a, b). Here, the visibility of the HOM interference is defined as *V* = *amplitude*/*average* = (*CC*_mean_ − *CC*_min_)/*CC*_mean_^20^. The triangle-shape of the HOM dip in Fig. 4(a, b) is caused by the group-velocity matching condition in PPKTP crystal at telecom wavelengths^21^,^22^. The widths of the dips in Fig. 4(a, b) are similar, around 1.33 mm (4.4 ps), since the width of the dip is determined by the length of the crystal^23^. The high visibilities in Fig. 4 confirmed the high indistinguishability of the generated photons.

**Figure 5:**
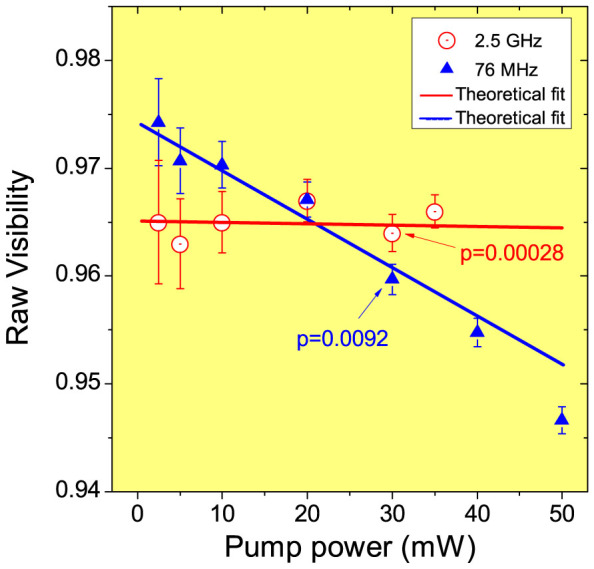
Raw visibilities versus pump powers for 2.5 GHz and 76 MHz lasers. At 30 mW, the average photon numbers per pulse were 0.00028 and 0.0092 for 2.5 GHz and 76 MHz lasers. The uncertainties of these visibilities were derived using Poissonian errors on the coincidence counts. The solid lines are the theoretical fit.

To compare the different performance of the 2.5 GHz and 76 MHz lasers, we repeated the HOM interference test at different pump powers. Without subtract any background counts, we compare the raw visibilities of HOM dip at different pump powers in Fig. 5. At a low pump power of 2.5 mW, the 76 MHz laser has a visibility of 97.4 ± 0.4%, slightly higher than the result by the 2.5 GHz laser, 96.5 ± 0.6%, since the 76 MHz laser has a better spectral profile. At 30 mW, the average photon numbers per pulse were 0.00028 and 0.0092 for 2.5 GHz and 76 MHz lasers. Note the average photon numbers per pulse in the HOM interference test were slightly higher than that in the ToA test, because we slightly improved the experimental condition in the test of HOM interference.

It is noteworthy that the visibilities by the 76 MHz laser decrease rapidly when the pump powers increase. In contrast, the visibilities by the 2.5 GHz laser shows almost no decrease up to 35 mW, the maximum SHG power we have achieved in experiment. This experimental result is also confirmed theoretically by directly calculating the evolution of the wave function of the state generated from the SPDC source. The theoretical model incorporate the practical imperfections including transmittance losses in channels (delay line, fiber connectors, etc) and detectors, and the mode-matching efficiency *η_M_* at the beam splitter. The losses are directly measured from our setup and *η_M_* is treated as a fitting parameter. Note that in the experiment, *η_M_* is close to unity and not easy to measure with enough accuracy while as shown in Supplementary Information, the HOM visibility is very sensitive to *η_M_*. In Fig. 5, the data are fitted with *η_M_* values of 0.9828 and 0.9878 for 2.5 GHz laser and 76 MHz laser, respectively. Higher *η_M_* value for the 76 MHz laser is reasonable, because the indistinguishablites of the twin photons generated by the 76 MHz laser is slightly better than that by the 2.5 GHz laser, which can be roughly checked by the spectra of the signal and idler in Fig. 2(c) and (d). After the transmittance efficiency and the mode-matching efficiency are fixed, the HOM visibilities are only determined by *p*, the average photon pairs per pulse (i.e., the generation probability of one pair per pulse). The low value *p* by the 2.5 GHz laser guarantees its high visibilities at high pump powers. In Fig. 5, we observe an excellent agreement with the experimental data and the theoretical lines. For the details of the theoretical model, see Supplementary Information. It is also worth to note that recently we have developed an alternative method to simulate this type of SPDC based experiments by using characteristic functions that have been often utilized in theory of continuous variable quantum optics^24^. This method is simpler than the direct computation of the wave functions and allows us to derive the closed form of the HOM dip including the above practical imperfections and all multi-photon effects.

## Discussion and Outlook

With the theoretical model (See the Supplementary Information), we further calculate the visibilities at high pump powers, as shown in Tab. 1. It is interesting to note that, at high pump power up to 3 W, the visibility by 76 MHz laser will decrease to 62.4%, while the 2.5 GHz laser still can keep the visibility higher than 90%, mainly thanks to the low average photon numbers per pulse. To experimentally demonstrate this high visibilities at high pump powers in the future, we could update the PPKTP bulk crystal to a PPKTP waveguide. Also, SHG power of the comb laser at 10 GHz repetition rate needs to be improved. See the Supplementary Information for the HOM interference of this comb laser at 10 GHz repetition rate. Nevertheless, our experimental results in Fig. 5 have clearly shown the non-degradation of HOM visibilites at high pump powers.

We notice that many other schemes have been reported to reduce the multi-pair emission. Broome *et al* demonstrated to reduce the multi-pair emission by temporally multiplexing the pulsed pump lasers by two times^18^. Ma *et al* tried to reduce such effects by multiplexing four independent SPDC sources^25^. All the previous methods have a limited effect, because the units they multiplexed were limited. If they increase the multiplexed units, the setup will be very complex. The GHz-repetition-rate-laser pumped photon source in our scheme provide a very simple and effective way to reduce the multi-pair emission. In addition, GHz repetition rate lasers are now commercially available^26^,^27^,^28^.

Therefore, our scheme will be a reasonable option to construct the next generation of twin photon sources with high brightness, low multi-pair emission and high detection efficiency. In the traditional twin photon source technologies, 76 MHZ pump laser is compatible with a BBO crystal (with maximum *p* ~ 0.06, corresponding to photon pair generation rate of 5 MHz) and Si avalanche photodiode (with acceptable maximal photon numbers of 5–10 MHz). In the next generation of twin photon sources, the 10 GHz laser should be combined with a high efficiency crystals, e.g., PPKTP crystal (or waveguide, with maximum *p* ~ 0.1, corresponding to photon pair generation rate of 1 GHz), and high speed detectors, e.g., the SNSPD (with acceptable maximal photon numbers of 25–100 MHz). Consequently, we expect more than tenfold brighter photon source in conjunction with both low multiple photon pairs production and high spectral purity. Further, a repetition tunability allow us to obtain an optimal generation probability in a pulse without sacrificing a photon counting rate.

## Conclusion

We have demonstrated a twin photon source pumped by a 10-GHz-repetition-rate tunable comb laser. The photons are generated from GVM-PPKTP crystal and detected by highly efficient SNSPDs. The SNR test and HOM interference test with 2.5 GHz laser showed a high SNR and high visibilities not degrading at high pump powers, much higher than that pumped by the 76 MHz laser. The high-repetition-rate pump laser, the GVM-PPKTP crystal, and the highly efficient detectors constitute a powerful tool box at the telecom wavelengths. We believe our tool box may have a variety of applications in the future quantum information and communication technologies.

## Methods

### The comb laser

The picosecond pulses from the comb laser are generated with the following principles^12^,^13^. A continuous-wave (cw) light emitted from a single-mode laser diode (LD) with a wavelength of around 1553 nm is led into a Mach-Zehnder-modulator (MZM) and is converted to a comb signal with 10 GHz in spacing and 300 GHz in bandwidth. The MZM is fabricated on a LiNbO_3_ crystal and is driven by a 10 GHz radio-frequency signal. Because the comb signal has linear chirp, it can be formed to a picosecond pulse train with a repetition rate of 10 GHz by chirp compensation using a single-mode fiber. The comb laser also includes a pulse picker, so that the repetition frequency of the pulse train can be changed in the range of 10–0.625 GHz. In this experiment, we keep the temporal width around 2.5 ps. For more details of this kind of comb lasers, see Refs. 29, 30. See the Supplementary Information for more spectral and temporal information of this comb laser.

### The SHG

Generating a high-power second harmonic light (SHG) is a key point in this experiment. Since the average power per pulse of the comb laser is very low, we choose a periodically poled lithium niobate wave guide (PPLN-WG) for SHG. We tested both 10 GHz and 2.5 GHz repetition rate lasers. We found the SHG power with 2.5 GHz repetition rate was more stable than that with 10 GHz repetition rate. Therefore, the data in this experiment are mainly obtained by using 2.5 GHz repetition rate. With the input 2.5 GHz repetition rate fundamental laser at a power of 500 mW, we obtained 42 mW SHG power. After filtered by several short-pass filters to cut the fundamental light, we finally achieved a net SHG power of 35 mW. The transmission loss of the PPLN-WG was around 50%. Fig. 2(b) is the spectrum of 776.5 nm SHG laser, measured by a spectrometer (SpectraPro-2300i, Acton Research Corp.). Interestingly, it can be noticed that the comb structure no-longer exists in the SHG spectrum, which may be caused by a sum-frequency-generation process.

### The SPDC

For SPDC, the nonlinear crystal used in this experiment is a PPKTP crystal, which satisfy the GVM condition at telecom wavelength^14^,^31^,^32^,^33^,^34^. Thanks to the GVM condition, the spectral purity is much higher at telecom wavelength than that at visible wavelengths^15^. This spectrally pure photon source is very useful for multi-photon interference between independent sources^35^,^36^,^37^. Figure 2(c, d) are the observed spectra of the signal and idler photons, measured by a spectrometer (SpctraPro-2500i, Acton Research Corp.). The FWHMs of the twin photons are about 1.2–1.3 nm, similar as the spectral width of the photons pumped by 76 MHz laser^14^.

### The SNSPDs

Our superconducting nanowire single photon detectors (SNSPDs) have a system detection efficiency (SDE) of around 70% with a dark count rate (DCR) less than 1 kcps^9^,^16^,^17^. The SNSPD also has a wide spectral response range that covers at least from 1470 nm to 1630 nm wavelengths^9^. The measured timing jitter and dead time (recovery time) were 68 ps^16^ and 40 ns^38^.

## Supplementary Material

Supplementary Information Supplementary

## Figures and Tables

**Table 1:** Visibilities at high pump powers. The estimated average photon pairs per pulse and the corresponding visibilites at different pump powers for 76 MHz and 2.5 GHz laser. The visibilities are directly determined by the average photon pairs per pulse.

	30 mW	~300 mW	~3 W
76 MHz	0.0092 (96.1%)	0.092 (86.1%)	0.92 (62.4%)
2.5 GHz	0.00028 (96.4%)	0.0028 (96.0%)	0.028 (91.8%)
