# Supplementary material for: Efficient generation of twin photons at telecom wavelengths with 2.5 GHz repetition-rate-tunable comb laser
Source: Sci Rep. 2014 Dec 19;4:7468. doi: 10.1038/srep07468 (PMC4650829; doi:10.1038/srep07468)
Supplement: Supplementary Information — Supplementary [file srep07468-s1.pdf]

Supplemental Material for
Efficient generation of twin photons at telecom wavelengths with 2.5 GHz repetition-rate tunable comb laser

Rui-Bo Jin, Ryosuke Shimizu, Isao Morohashi, Kentaro Wakui, Masahiro Takeoka, Shuro Izumi, Takahide Sakamoto, Mikio Fujiwara, Taro Yamashita, Shigehito Miki, Hirotaka Terai, Zhen Wang, and Masahide Sasaki

Supplementary-I

In this part we provide more information of the comb laser at 10 GHz and 2.5 GHz repetition rates. Figure 1 compares the spectra, autocorrelation, and temporal sequences of the comb laser at 10 GHz and 2.5 GHz repetition rates. Figure 2 shows the Hong-Ou-Mandel dip for the comb laser at 10 GHz, with a similar bandwidth and visibility as the results by the laser at 2.5 GHz repetition rate.

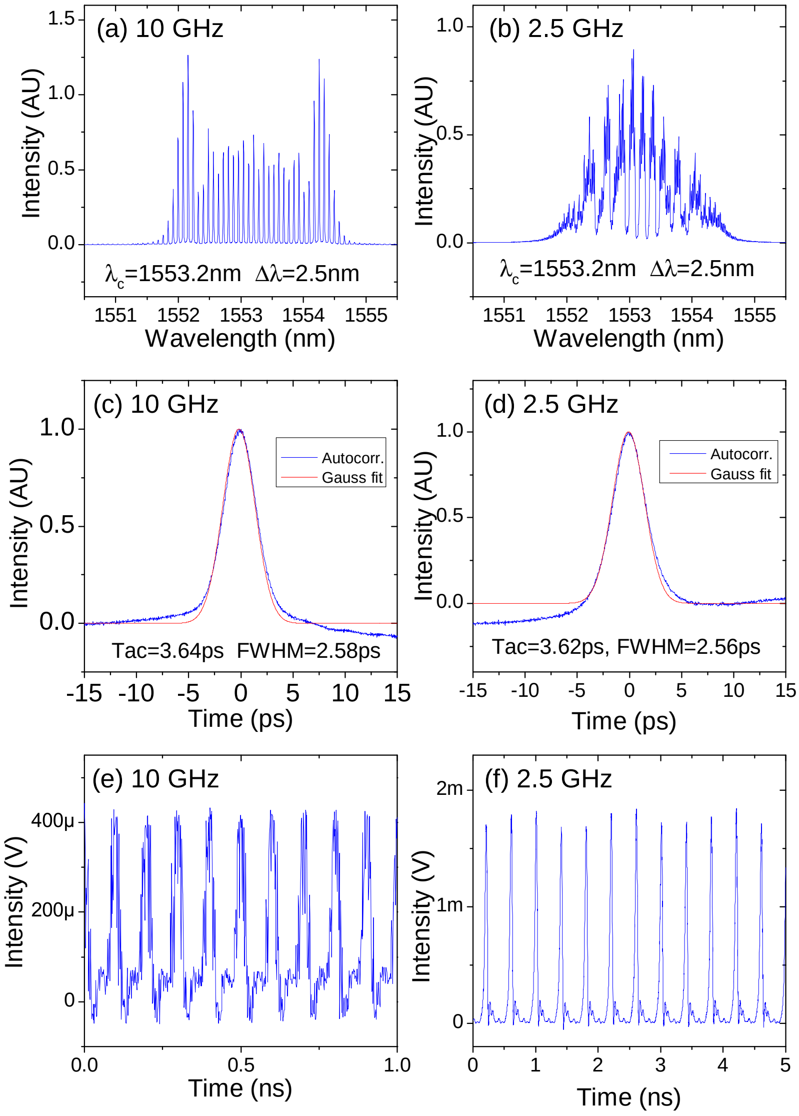

FIG. 1: (a-f) The spectra, autocorrelation, and temporal sequences of the comb laser at 10 GHz and 2.5 GHz repetition rates. The full-width-at-half-maximum (FWHM) of the autocorrelation data are round 3.6 ps, corresponding to FWHM of 2.6 ps for the fundamental lasers.

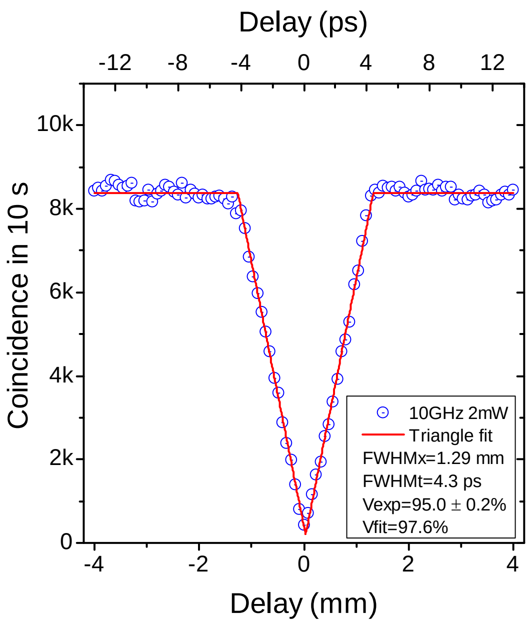

FIG. 2: Hong-Ou-Mandel dip for comb laser at 10 GHz repetition rate, fitted with triangle shape function. The pump power is 2 mW.

Supplementary-II

In this part we investigate the relationship between signal to noise ratio (SNR) and the main/side peaks in Time of Arrival (ToA) data. The output state from the spontaneous parametric down conversion (SPDC) can be expressed as

$$|\psi\rangle = \sqrt{1-\lambda^2}\sum_{n=0}^{\infty}\lambda^n |n,n\rangle = \sqrt{1-\lambda^2}(|0,0\rangle + \lambda|1,1\rangle + \lambda^2|2,2\rangle + ...), \tag{1}$$

where λ is the squeezing parameter. The probability for the n-pair photons per pulse is

$$Pr_n = (1-\lambda^2)\lambda^{2n}. \tag{2}$$

The average number of photon pairs per pulse is

$$p = (1-\lambda^2)\sum_{n=0}^{\infty} n\lambda^{2n} = \frac{\lambda^2}{1-\lambda^2}. \tag{3}$$

The 1-pair photons ($|1,1\rangle$ components in Eq. (1)) are the "*signal*", e.g., for constituting a single-photon source. All the other photons are contamination, and should be viewed as the "*noise*". Therefore, the signal to noise ratio (SNR) can be naturally defined as the ratio of single pair emission rate over all the other n-pair emission rates,

$$SNR \equiv \frac{Pr_1}{Pr_2 + Pr_3 + Pr_4 + ...} = \frac{(1-\lambda^2)\lambda^2}{(1-\lambda^2)(\lambda^4+\lambda^6+\lambda^8+...)} = \frac{1}{\sum_{n=1}^{\infty}\lambda^{2n}} = \frac{1-\lambda^2}{\lambda^2} = \frac{1}{p}. \tag{4}$$

In Eq. (4), it can be noticed that the SNR is the inverse of the averaged photon pair number per pulse, p. For a low pump power, where $\lambda^2 \ll 1$,

$$SNR = \frac{1-\lambda^2}{\lambda^2} \approx \frac{1}{\lambda^2}. \tag{5}$$

In the realistic situation, it may be difficult to estimate the SNR by using the experimental data. In contrast, here we provide a new method that enables us to simply evaluate the SNR from the main/side peaks in the experimental

ToA data. In the following calculation, we assume the pump power is very small so that $\lambda^2 \ll 1$ is satisfied. We can omit all the higher order terms in Eq. (1), and only consider the 0-, 1-, and 2-pair emissions. In the ToA data, the probability of the main peak (P_{main}) is proportional to the 1,1 click probability from total emission.

$$P_{main} \approx (1-\lambda^2)\lambda^2\eta^2 + (1-\lambda^2)\lambda^4(1-(1-\eta)^2)^2 = (1-\lambda^2)\lambda^2\eta^2[1+\lambda^2(2-\eta)^2], \tag{6}$$

where we only consider the 1- and 2-pair emission. η is the overall detection efficiency for the signal and idler photons. Assuming the dead time of the detector (~40 ns in our experiment) is longer than the peak-to-peak interval of the pump laser (~13 ns in our experiment), the probability of the side peak (P_{side}) is proportional to the 1, 0 click probability at the main peak position ($P_{1,0m}$, 1-click in start channel, and 0-click in stop channel), multiplied by the 1 click probability at the side peak position (P_{1s}, 1-click in stop channel).

$$P_{1,0m} \approx (1-\lambda^2)\lambda^2\eta(1-\eta) + (1-\lambda^2)\lambda^4(1-(1-\eta)^2)(1-\eta)^2 = (1-\lambda^2)\lambda^2\eta(1-\eta)[1+\lambda^2(2-\eta)(1-\eta)]. \tag{7}$$

$$P_{1s} \approx (1-\lambda^2)\lambda^2\eta + (1-\lambda^2)\lambda^4(1-(1-\eta)^2) = (1-\lambda^2)\lambda^2\eta[1+\lambda^2(2-\eta)]. \tag{8}$$

$$P_{side} = P_{1,0m} \times P_{1s} \approx (1-\lambda^2)^2\lambda^4\eta^2(1-\eta) \times [1+\lambda^2(2-\eta)][1+\lambda^2(2-\eta)(1-\eta)]. \tag{9}$$

Therefore, we can evaluate [(main peak − side peak)/side peak] as

$$\begin{aligned} \frac{\text{main peak}-\text{side peak}}{\text{side peak}} &= \frac{P_{main}-P_{side}}{P_{side}} \approx \frac{(1-\lambda^2)\lambda^2\eta^2[1+\lambda^2(2-\eta)^2]-(1-\lambda^2)^2\lambda^4\eta^2(1-\eta)[1+\lambda^2(2-\eta)][1+\lambda^2(2-\eta)(1-\eta)]}{(1-\lambda^2)^2\lambda^4\eta^2(1-\eta)[1+\lambda^2(2-\eta)][1+\lambda^2(2-\eta)(1-\eta)]} \\ &\approx \frac{(1-\lambda^2)\lambda^2\eta^2-(1-\lambda^2)^2\lambda^4\eta^2(1-\eta)}{(1-\lambda^2)^2\lambda^4\eta^2(1-\eta)} \approx \frac{\lambda^2\eta^2-\lambda^4\eta^2(1-\eta)}{\lambda^4\eta^2(1-\eta)} \approx \frac{\lambda^2\eta^2}{\lambda^4\eta^2(1-\eta)} = \frac{1}{\lambda^2(1-\eta)} \propto \frac{1}{\lambda^2}. \end{aligned} \tag{10}$$

In Eq. (10), the approximations were achieved by assuming $\lambda^2 \ll 1$. By comparing Eq. (5) and Eq. (10), we can learn that Eq.(5) can be used to approximate the SNR. Therefore, it is reasonable to calculate the SNR in a logarithmic scale as

$$SNR \approx 10log_{10}[(\text{main peak} - \text{side peak})/\text{side peak}] \approx 10log_{10}[\frac{1}{\lambda^2(1-\eta)}] \approx 10log_{10}[\frac{1}{\lambda^2}]. \tag{11}$$

The last approximation is valid if η is sufficiently low.

In the SNR test in this experiment, at 30 mW pump power, the overall detection efficiencies (η) were estimated as 0.31 for 76 MHz laser and 0.30 for 2.5 GHz laser; the average photon numbers per pulse (p) were estimated as 0.0079 for 76 MHz laser and 0.00021 for 2.5 GHz laser; $\lambda^2 = p/(1+p)$ were estimated as 0.0078 for 76 MHz laser and 0.00021 for 2.5 GHz laser. Therefore, the condition of $\lambda^2 \ll 1$ is fully satisfied in the experiment. In Eq.(11), with $\eta = 0.31$, $10log_{10}[\frac{1}{\lambda^2(1-\eta)}] = 10log_{10}[\frac{1}{\lambda^2}] + 10log_{10}[\frac{1}{(1-\eta)}] \approx 10log_{10}[\frac{1}{\lambda^2}] + 1.61$. While $10log_{10}[\frac{1}{\lambda^2}] \approx 21.08$ for $\lambda^2 = 0.0078$.

Supplementary-III

In this part, we numerically analyze the relationship between photon-pair generation rate (i.e., average photon pair per pulse) and HOM interference visibility.

The model

Here, we describe a numerical model of the HOM experiment. The model is described in Fig. 3(a) (without delay) and (b) (with delay) where $\eta_{A,B}$ represent transmittances of mode A and B (losses are effectively described by beam splitters), respectively, and η_{D_1} and η_{D_2} are the detector efficiencies. The mode mismatch between the signal and idler pulses is directly reflected to the HOM interference visibility. In general, the signal and idler pulses occupy slightly different modes in frequency, time, or spatial degrees of freedom. This is phenomenologically modeled by introducing two virtual beam splitters with transmittance η_M (which directly corresponds to the mode matching efficiency) that split the signal and idler into three modes, overlapped part (A and B) and unoverlapped parts occupied by the signal (E) and the idler (F) (see Fig. 3(a)).

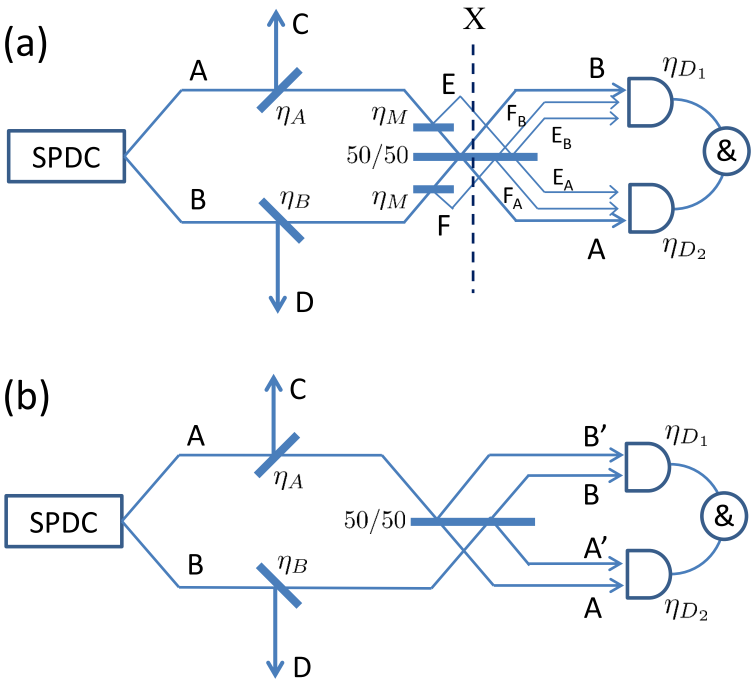

FIG. 3: Models. (a) No delay (HOM dip). The mode mismatch at the 50/50 beam splitter is represented by the beam splitters η_M. (b) With delay. The delay is represnted by the spatial difference at the 50/50 beam splitter.

The HOM interference visibility is defined as

$$V = \frac{CC_{\text{mean}} - CC_{\text{min}}}{CC_{\text{mean}}}, \tag{12}$$

where CC_{min} and CC_{mean} are the coincidence count rates with zero-delay and large delay (i.e., with and without interference between the signal and idler), respectively. In the following we derive CC_{min} and CC_{mean} separately from our model.

The initial state from the SPDC source is given by a two-mode squeezed-vacuum state

$$|\psi\rangle_{AB} = \sqrt{1-\lambda^2}\sum_{n=0}^{\infty}\lambda^n|n\rangle_A|n\rangle_B, \tag{13}$$

where λ is the squeezing parameter, and $\lambda^2/(1-\lambda^2) = p$ is the average photon pairs per pulse. Let $\hat{V}^{\eta}_{A(B)}$ be a beam splitting operator on mode $A(B)$ with transmittance η which transforms the photon number states $|n_1\rangle|n_2\rangle$ as

$$\begin{aligned}\hat{V}^{\eta}_{AB}|n_1\rangle|n_2\rangle \;&=\; \frac{1}{\sqrt{n_1!n_2!}}\sum_{k_1=0}^{n_1}\sum_{k_2=0}^{n_2}\binom{n_1}{k_1}\binom{n_2}{k_2}(-1)^{k_2}\\ &\times\sqrt{\eta}^{\,n_2+k_1-k_2}\sqrt{1-\eta}^{\,n_1-k_1+k_2}\\ &\times\sqrt{(k_1+k_2)!(n_1+n_2-k_1-k_2)!}\\ &\times|k_1+k_2\rangle\,|n_1+n_2-k_1-k_2\rangle\,.\end{aligned} \tag{14}$$

Applying the beam splitters $\hat{V}^{\eta_A}_{AC}$, $\hat{V}^{\eta_B}_{BD}$, $\hat{V}^{\eta_M}_{AE}$, $\hat{V}^{\eta_M}_{BF}$, and $\hat{V}^{1/2}_{AB}$ onto the two-mode squeezed vacuum (state at X in Fig. 3(a)), we obtain

$$
\begin{aligned}
&\hat{V}_{AB}^{1/2}\hat{V}_{BF}^{\eta_M}\hat{V}_{AE}^{\eta_M}\hat{V}_{BD}^{\eta_B}\hat{V}_{AC}^{\eta_A}|\psi\rangle_{AB}|0\rangle_C|0\rangle_D|0\rangle_E|0\rangle_F \\
&= \sqrt{1-\lambda^2}\sum_{n=0}^{\infty}\lambda^n\sum_{k_1=0}^{n}\binom{n}{k_1}^{1/2}\eta_A^{k_1/2}(1-\eta_A)^{\frac{n-k_1}{2}}\sum_{k_2=0}^{n}\binom{n}{k_2}^{1/2}\eta_B^{k_2/2}(1-\eta_B)^{\frac{n-k_2}{2}} \\
&\quad\times\sum_{k_3=0}^{k_1}\binom{k_1}{k_3}^{1/2}\eta_M^{k_3/2}(1-\eta_M)^{\frac{k_1-k_3}{2}}\sum_{k_4=0}^{k_2}\binom{k_2}{k_4}^{1/2}\eta_M^{k_4/2}(1-\eta_M)^{\frac{k_2-k_4}{2}} \\
&\quad\times\sum_{k_5=0}^{k_3}\sum_{k_6=0}^{k_4}\binom{k_3}{k_5}\binom{k_4}{k_6}\left(\frac{1}{2}\right)^{\frac{k_3+k_4}{2}}(-1)^{k_6}\left(\frac{(k_5+k_6)!\,(k_3+k_4-k_5-k_6)!}{k_3!\,k_4!}\right)^{1/2} \\
&\quad\times|k_5+k_6\rangle_A|k_3+k_4-k_5-k_6\rangle_B|n-k_1\rangle_C|n-k_2\rangle_D|k_1-k_3\rangle_E|k_2-k_4\rangle_F \\
&= \sqrt{1-\lambda^2}\sum_{n=0}^{\infty}\lambda^n\sum_{k_1=0}^{n}\binom{n}{k_1}^{1/2}\eta_A^{k_1/2}(1-\eta_A)^{\frac{n-k_1}{2}}\sum_{k_2=0}^{n}\binom{n}{k_2}^{1/2}\eta_B^{k_2/2}(1-\eta_B)^{\frac{n-k_2}{2}} \\
&\quad\times\sum_{k_3=0}^{k_1}\binom{k_1}{k_3}^{1/2}\eta_M^{k_3/2}(1-\eta_M)^{\frac{k_1-k_3}{2}}\sum_{k_4=0}^{k_2}\binom{k_2}{k_4}^{1/2}\eta_M^{k_4/2}(1-\eta_M)^{\frac{k_2-k_4}{2}}\left(\frac{1}{2}\right)^{k_3+k_4} \\
&\quad\times\sum_{l=0}^{k_3+k_4}\sum_{k_5=\max\{0,l-k_4\}}^{\min\{l,k_3\}}(-1)^{l-k_5}\left\{\binom{k_3}{k_5}\binom{k_4}{l-k_5}\binom{l}{k_5}\binom{k_3+k_4-l}{k_3-k_5}\right\}^{1/2} \\
&\quad\times|l\rangle_A|k_3+k_4-l\rangle_B|n-k_1\rangle_C|n-k_2\rangle_D|k_1-k_3\rangle_E|k_2-k_4\rangle_F,
\end{aligned}
\tag{15}
$$

where $l = k_5 + k_6$ and we have used the relation

$$
\binom{k_3}{k_5}\binom{k4}{k_6}\left(\frac{(k_5+k_6)!\,(k_3+k_4-k_5-k_6)!}{k_3!\,k_4!}\right)^{1/2} = \left\{\binom{k_3}{k_5}\binom{k4}{k_6}\binom{k_5+k_6}{k_5}\binom{k_3+k_4-k_5-k_6}{k_3-k_5}\right\}^{1/2}. \tag{16}
$$

Note that $\hat{V}^{1/2}$ should be applied to mode E and F, which will be discussed later. From Eq. (15) we find the joint probability of having l, k_3+k_4-l, $n-k_1$, $n-k_2$, k_1-k_3, k_2-k_4 photons in mode A-F at X:

$$
\begin{aligned}
&P_{ABCDEF}^{X}(l,k_3+k_4-l,n-k_1,n-k_2,k_1-k_3,k_2-k_4) \\
&= (1-\lambda)^2\lambda^{2n}\eta_A^{k_1}(1-\eta_A)^{n-k_1}\eta_B^{k_2}(1-\eta_B)^{n-k_2}\eta_M^{k_3+k_4}(1-\eta_M)^{k_1+k_2-k_3-k_4}\left(\frac{1}{2}\right)^{k_3+k_4}\binom{n}{k_1}\binom{n}{k_2}\binom{k_1}{k_3}\binom{k_2}{k_4} \\
&\times\left\{\sum_{k_5=\max\{0,l-k_4\}}^{\min\{l,k_3\}}(-1)^{l-k_5}\left\{\binom{k_3}{k_5}\binom{k_4}{l-k_5}\binom{l}{k_5}\binom{k_3+k_4-l}{k_3-k_5}\right\}^{1/2}\right\}^2.
\end{aligned}
\tag{17}
$$

The 50/50 beam splitting of mode E (F) into E_A and E_B (F_A and F_B) adds extra binomial distribution terms $\binom{k_1-k_3}{k_7}\binom{k_2-k_4}{k_8}\left(\frac{1}{2}\right)^{k_1+k_2-k_3-k_4}$ to Eq. (17). The joint probability distribution for the state at the detectors is thus given by

$$
\begin{aligned}
&P_{ABCDE_AF_AE_BF_B}(l,k_3+k_4-l,n-k_1,n-k_2,k_7,k_2-k_4-k_8,k_1-k_3-k_7,k_8) \\
&= (1-\lambda)^2\lambda^{2n}\eta_A^{k_1}(1-\eta_A)^{n-k_1}\eta_B^{k_2}(1-\eta_B)^{n-k_2}\eta_M^{k_3+k_4}(1-\eta_M)^{k_1+k_2-k_3-k_4}\left(\frac{1}{2}\right)^{k_1+k_2}\binom{n}{k_1}\binom{n}{k_2}\binom{k_1}{k_3}\binom{k_2}{k_4} \\
&\times\binom{k_1-k_3}{k_7}\binom{k_2-k_4}{k_8}\left\{\sum_{k_5=\max\{0,l-k_4\}}^{\min\{l,k_3\}}(-1)^{l-k_5}\left\{\binom{k_3}{k_5}\binom{k_4}{l-k_5}\binom{l}{k_5}\binom{k_3+k_4-l}{k_3-k_5}\right\}^{1/2}\right\}^2.
\end{aligned}
\tag{18}
$$

The coincidence rate $CC_{\min}$ is then obtained by the sum of the joint probability:

$$
\begin{aligned}
CC_{\min} &= \sum_{n=0}^{\infty}\sum_{k_1=0}^{n}\sum_{k_2=0}^{n}\sum_{k_3=0}^{k_1}\sum_{k_4=0}^{k_2}\sum_{k_7=0}^{k_1-k_3}\sum_{k_8=0}^{k_2-k_4}\sum_{l=0}^{k_3+k_4}\left\{1-(1-\eta_{D_1})^{l+k_2-k_4+k_7-k_8}\right\}\left\{1-(1-\eta_{D_2})^{-l+k_1+k_4-k_7+k_8}\right\} \\
&\quad\times P_{ABCDE_AF_AE_BF_B}(l,k_3+k_4-l,n-k_1,n-k_2,k_7,k_2-k_4-k_8,k_1-k_3-k_7,k_8).
\end{aligned}
\tag{19}
$$

The derivation of CC_{mean} is rather simple since there is no interference at the 50/50 beam splitter due to the delay. This is illustrated in Fig. 3(b). Note that we do not need η_M. The two-mode squeezed vacuum from the SPDC source has a joint photon distribution:

$$P_{AB}(n,n) = (1-\lambda^2)\lambda^{2n}. \tag{20}$$

The beam splitting operation simply spread this distribution in a binomial manner. For example, after the beam splitter η_A, the joint distribution is given by

$$P_{ABC}(n,k_1,n-k_1) = (1-\lambda^2)\lambda^{2n}\binom{n}{k_1}\eta_A^{k_1}(1-\eta_A)^{n-k_1}. \tag{21}$$

Applying the η_B and 50/50 beam splitters in a similar way, we have

$$\begin{aligned} &P_{AA'BB'CD}(k_3,k_2-k_4,k_4,k_1-k_3,n-k_1,n-k_2) \\ &= (1-\lambda^2)\lambda^{2n}\binom{n}{k_1}\binom{n}{k_2}\binom{k_1}{k_3}\binom{k_2}{k_4}\eta_A^{k_1}(1-\eta_A)^{n-k_1}\eta_B^{k_2}(1-\eta_B)^{n-k_2}\left(\frac{1}{2}\right)^{k_1+k_2}, \end{aligned} \tag{22}$$

before the detectors. The coincidence count CC_{mean} is then given by

$$\begin{aligned} CC_{\text{mean}} \;=\; &\sum_{n=0}^{\infty}\sum_{k_1=0}^{n}\sum_{k_2=0}^{n}\sum_{k_3=0}^{k_1}\sum_{k_4=0}^{k_2}\left\{1-(1-\eta_{D_1})^{k_2+k_3-k_4}\right\}\left\{1-(1-\eta_{D_2})^{k_1-k_3+k_4}\right\} \\ &\times P_{AA'BB'CD}(k_3,k_2-k_4,k_4,k_1-k_3,n-k_1,n-k_2). \end{aligned} \tag{23}$$

The HOM visibility in Eq. (12) is thus calculable from Eqs. (19) and (23).

Numerical result

The transmittances (efficiencies) of each components in the experiment are summarized in Table I (see Fig. 3 for the theoretical model and the corresponding experimental setup in Main text. In fact, the HOM visibility is extremely sensitive to the mode matching factor η_M. It is however not easy to estimate the mode matching factor η_M experimentally with enough accuracy.

In Fig. 4, we plot the numerical results with various η_M, and the experimental data with the 76 MHz laser. The experimental average photon-pair p is estimated from the experimental count rates. The experimental data fit the theoretical lines well within $0.9848 \le \eta_M \le 0.9888$. With the parameters in Table I, we also calculated the performance of our scheme at high photon-pair generation rate, as shown in Fig. 5 and Table II. From this simulation, we find several interesting relationship. (1). The visibility is directly determined by the average photon-pairs. (2). The slope of this line is very sensitive to the unbalanced loss in the delay arm and non-delay arm. (3). The Y-intercept of this line very sensitive to the mode matching efficiency.

TABLE I: Transmittance and efficiency of the components in the experiment. SMFC: single mode fiber coupler. FC: fiber connector. SNSPD: superconducting nanowier single photon detector.

η_A	0.42	SMFC + FCs
η_B	0.29	SMFC + FCs + Delay line
η_{D_1}	0.68	SNSPD1
η_{D_2}	0.70	SNSPD2

TABLE II: The visibilities at different photon-pair generation rate.

p	0.001	0.005	0.01	0.05	0.1	0.2	0.5	1	2
V	0.974	0.968	0.960	0.906	0.854	0.781	0.677	0.618	0.585

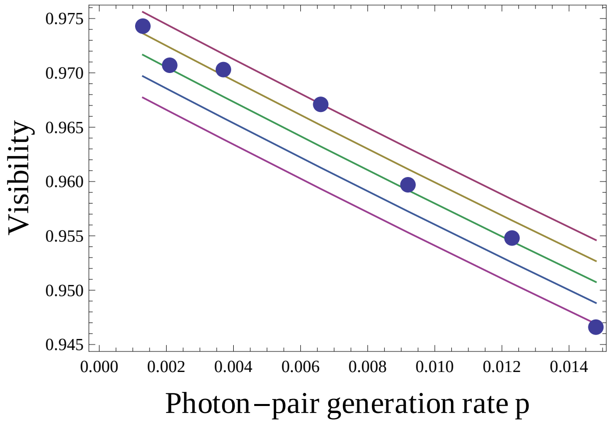

FIG. 4: The HOM visibility versus p. The solid lines represent theoretical curves with $\eta_M = 0.9888, 0.9878, 0.9868, 0.9858, 0.9848$ from the top to the bottom. The plots are the experimental results with the 76 MHz laser.

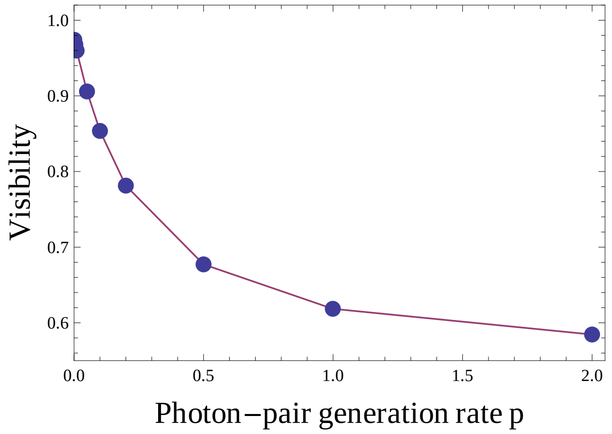

FIG. 5: The HOM visibilities at different p values, with $\eta_M = 0.9878$.
